# Relationship between Respiratory Load Perception and Perception of Nonrespiratory Sensory Modalities in Subjects with Life-Threatening Asthma

**DOI:** 10.1155/2012/310672

**Published:** 2012-06-13

**Authors:** Kathleen L. Davenport, Chien Hui Huang, Matthew P. Davenport, Paul W. Davenport

**Affiliations:** ^1^Department of Rehabilitation Medicine, University of Washington, 1959 Northeast Pacific Street, P.O. Box 356490, Seattle, WA 98195, USA; ^2^Department of Physical Therapy, Tzu Chi University, 701 Zhongyang Road, Section 3, Hualien 97004, Taiwan; ^3^Department of Chemistry and Food Science, Framingham State University, 100 State Street, Framingham, MA 01701, USA; ^4^Department of Physiological Sciences, University of Florida, P.O. Box 100144 HSC, Gainesville, FL 32610, USA

## Abstract

Subjects with life-threatening asthma (LTA) have reported decreased sensitivity to inspiratory resistive (*R*) loads. It is unknown if decreased sensitivity is specific for inspiratory *R* loads, other types of respiratory loads, or a general deficit affecting sensory modalities. This study hypothesized that impairment is specific to respiratory stimuli. This study tested perceptual sensitivity of LTA, asthmatic (A), and nonasthmatic (NA) subjects to 4 sensory modalities: respiratory, somatosensory, auditory, visual. Perceptual sensitivity was measured with magnitude estimation (ME): respiratory loads ME, determined using inspiratory *R* and pressure threshold (PT) loads; somatosensory ME, determined using weight ranges of 2–20 kg; auditory ME, determined using graded magnitudes of 1 kHz tones delivered for 3 seconds bilaterally; visual ME, determined using gray-to-white disk intensity gradations on black background. ME for inspiratory *R* loads lessened for LTA over A and NA subjects. There was no significant difference between the 3 groups in ME for PT inspiratory loads, weight, sound, and visual trials. These results demonstrate that LTA subjects are poor perceivers of inspiratory *R* loads. This deficit in respiratory perception is specific to inspiratory *R* loads and is not due to perceptual deficits in other types of inspiratory loads, somatosensory, auditory, or visual sensory modalities.

## 1. Introduction

 Asthma is a respiratory disease frequently diagnosed in childhood. To control and/or prevent an asthma attack, it is important for the patient to heed initial symptoms and to be compliant with their prescribed medication(s). Failure to recognize and self-manage of an asthma exacerbation is one cause of life-threatening asthma (LTA) [1–3]. Difficulty in perceiving asthma symptoms can be one of many factors causing the patient to fail to recognize the onset of an asthma attack [2, 4, 5]. A subpopulation of asthmatic patients with a history of LTA has been reported with reduced perception of both intrinsic and extrinsic respiratory loads [2, 4]. These LTA asthmatic patients have an increased threshold for detection of inspiratory resistive loads, a decreased ability to scale the magnitude of inspiratory loads and a decreased perception of intrinsic bronchoconstriction [2, 4]. It has also been reported that the somatosensory cortex is not activated by inspiratory loads in these LTA subjects suggesting a sensory neural deficit in respiratory information processing in this subpopulation of asthmatic patients [1]. While it is evident that these LTA subjects have poor perception of respiratory mechanical loads, it is unknown if this is a specific deficit for respiratory mechanosensation or a general sensory perception deficit.

 Perception of respiratory stimuli has been studied by asking patients to estimate various respiratory load magnitudes and types using the modified Borg scale, visual analog scale, or cross-modality matching [1–4, 6–24]. In order to successfully perform the load perception task, the subject must attend to the load, sense the magnitude of the load, and then provide an estimate of their sense of load magnitude using scaling techniques. These studies have shown that adults and children are capable of estimating inspiratory resistive load magnitudes [1–4, 6–24]. This load magnitude estimation (ME) technique has also been used in multiple sensory modalities to investigate the perceptual sensitivity in subjects to various stimuli such as light [25], sound [26–29], and weight [21, 30–32]. The perceptual sensitivity to respiratory loads is similar to other somatosensory stimuli [21] in normal subjects. However, it is unknown if subjects with poor perception of respiratory loads also have poor perception of other sensory modalities or if the load perception deficit is specific to respiratory information processing.

 LTA patients have been shown to have a reduced detection and magnitude estimation of inspiratory resistive loads [2, 4]. It is unknown if this is specific to respiratory mechanosensation, specific to all somatosensation, or a general perceptual deficit. This study was designed to test the sensory perception of LTA subjects to respiratory, somatosensory, auditory, and visual stimuli. Respiratory perception was tested by two types in inspiratory mechanical loads, resistive loads, and pressure threshold loads. We reasoned that if LTA asthmatics have a general respiratory perception deficit, they would be poor perceivers of both types of loads. If LTA patients have a respiratory perception deficit that includes a somatosensory deficit, then they should have reduced perception of both respiratory and weight lifting magnitude estimation. If the LTA asthmatics have a respiratory perception deficit that includes a general sensory perception deficit, then they should have reduced perception of respiratory stimuli and arm weight, auditory, and visual stimuli would be expected. It was hypothesized that asthmatic patients with a history of LTA and a perception deficit of resistive respiratory stimuli exclusively will have unimpaired somatosensory, auditory, and visual perception. This hypothesis was tested in nonasthmatic, LTA asthmatics, and asthmatics without a history of LTA.

## 2. Methods

### 2.1. Subjects

 Three groups of subjects were tested in this study: (1) subjects (*n* = 7) with life-threatening asthma (LTA), (2) subjects (*n* = 10) with stable asthma (A), and (3) nonasthmatic (NA) subjects (*n* = 9). The subject ages in all groups ranged between 11 and 25 years. Mean ages were 16.3 ± 3.0 years for the LTA group, 17.2 ± 3.0 years for the A group, and 19.0 ± 4.2 years for the NA group. All A and LTA subjects were followed at the Pediatric Pulmonary Clinic at Shands Hospital, University of Florida, Gainesville, FL. The diagnosis of asthma was made by a pediatric pulmonologist, based on the American Thoracic Society criteria [33]. The University of Florida, Health Science Center, Institutional Review Board reviewed and approved this study. All participation was voluntary, and all subjects and the parents of minor subjects received informed consent.

 The LTA subjects were asymptomatic without exacerbation of their asthma within 4 weeks of the study. All LTA subjects had been admitted to the Pediatric Intensive Care unit with acute respiratory failure within the last four years. Following their life-threatening event, the LTA subjects were stabilized and maintained with inhaled corticosteroids and theophylline. The A subjects had moderate-to-severe asthma and were on daily maintenance treatment to control their asthma symptoms. However, they had never been admitted to an intensive care unit for respiratory failure. Both A and LTA groups used albuterol by metered dose inhaler (MDI) or by nebulizer as needed. The NA subjects were free of chronic respiratory disease. All subjects were free of any acute respiratory disease at least four weeks prior to the study. No subjects were hearing impaired or were visually impaired to the extent that contact lenses or glasses could not accurately correct their eye sight. Any subject requiring glasses or contact lenses wore them during all testing.

### 2.2. Pulmonary Function Tests

 A pulmonary function test (PFT) was administered after consent was obtained and before testing began. FEV_1_, FVC, FEV_1_/FVC, and resistance by the forced oscillation method were measured. Any subject with a baseline FEV_1_ less than 70% of predicted was eliminated from further participation in the study. No subject had a baseline FEV_1_, FEV_1_/FVC, or FVC lower than 70% of predicted, according to the American Thoracic Society standards.

### 2.3. Inspiratory Resistive Load Magnitude Estimation

 Perception of extrinsic respiratory loads was determined using magnitude estimation of inspiratory resistive loads with a modified Borg scale [1, 9, 11]. The subject was seated conformably in a sound isolated chamber, separated from the investigator and the experimental apparatus. The subjects had their nose clamped and respired through a mouthpiece connected to a non-rebreathing valve (Hans Rudolph, Kansas City, MO). Care was taken to suspend the valve to eliminate the need for the subject to bite the mouthpiece yet maintain an airtight seal.

 The resistive loads were sintered bronze disks placed in series in a loading manifold with stoppered ports between the disks [1]. The loading manifold was connected to a pneumotachograph by reinforced tubing to the inspiratory port of the non-rebreathing valve. The loading manifold was hidden from the subject's view. Mouth pressure (*P*_*M*_) was recorded from a port in the center of the non-rebreathing valve. *P*_*M*_ was sensed with a differential pressure transducer and a signal conditioner. Inspiratory airflow (*V*_*I*_′) was recorded with a differential pressure transducer and signal conditioner connected to the pneumotachograph. The *V*_*I*_′ was integrated to obtain the inspired volume (*V*_*I*_). The *P*_*M*_, *V*_*I*_, and *V*_*I*_′ were recorded on a polygraph. The *V*_*I*_′ was also displayed on an oscilloscope placed in front of the subjects, which they used to target their breathing during the study. Resistances were selected by removing a stopper and allowing the subject a single inspiration through the selected port. They were monitored with a digital video camera throughout the study.

 Before testing began, the peak *V*_*I*_′ during normal, tidal breathing was determined and displayed as a horizontal line on the oscilloscope. The subject was allowed to practice *V*_*I*_′ targeting prior to the load practice session. The load practice session consisted of a series of test loads, including a low and high load, presented with a verbal cue (“small” versus “large,” resp.) to familiarize the subject with the range of loads.

 Standard 10-point category Borg scale rating was the modality used by subjects to estimate magnitude of the external respiratory load. Subjects were asked to press a button on a battery-powered device that corresponded to the Borg scale rating for the test breath. The voltage from the device was displayed on the polygraph and calibrated to the corresponding Borg scale rating.

 The presentation of the resistive loads for the magnitude estimation was divided into two experiment trials. Both trials consisted of six resistive load magnitudes (1.64, 2.48, 3.26, 6.95, 11.46, and 20.48 cm H_2_O/L/s) and no-load presented five times each in randomized block order, as described previously [2]. Subjects were given a 5–10-minute break between each trial. Thus, each subject was exposed to each load magnitude a total of 10 times. Subjects were given a cue (red light) to signal that the next breath was a test breath, which the subject must estimate. The subject inspired to the target *V*_*I*_′ on each breath (control and test). The subject made the estimate immediately after the test breath using the Borg scale. Three to six unloaded breaths separated each test breath.

### 2.4. Inspiratory Pressure Threshold Load Magnitude Estimation

 Perception of pressure threshold (PT) loads was administered in the same fashion as the inspiratory resistive loads [16]. The same mouthpiece, Borg scale, and methods were used for this portion of the study. The only difference was the loading manifold, and therefore a theoretical difference in perception of the respiratory load. This loading manifold included spring-loaded pressure-threshold valves with stoppered ports over each valve. The valves would open when a calibrated, specific inspiratory pressure was achieved. The PT load magnitude was the inspiratory pressure required to open the valve allowing air to flow. There was a PT load practice session before this test to familiarize subjects with the respiratory loads. The PT load practice session consisted of a series of test loads, including a low and high load, presented after a verbal cue (“small” versus “large,” resp.). The presentation of the PT loads for the ME was divided into two experiment trials. Both trials consisted of 7 PT load magnitudes (2.35, 4.12, 5.22, 10.27, 18.80, 23.14, and 27.45 cm H_2_O) and no-load were presented five times each in a randomized block order. Thus, each subject was exposed to each PT load ten times. Subjects were given a 5–10-minute break in between each trial.

### 2.5. Weight Magnitude Estimation

Subjects were seated conformably in a sound isolated chamber, separated from the investigator and the experimental apparatus. They placed the elbow of their dominant hand on an armrest of a chair. They placed their forearm vertically so their hands were raised in the air and grasped a handle which was attached to a rope. The rope was connected through a 2-pulley system to a bucket, where weights were added out of the subjects' view. Once subjects had gripped the handle comfortably, the rope was pulled taught so there was no slack in the rope.

 Weights (5, 15, 30, 60, and 80 ounces) and no-weight were placed in the bucket out of view of the subjects. Subjects were then cued by a red light to lower their forearm directly down onto the armrest to sense the weight. Another investigator was seated in the room with the subjects during the experiment. After subjects pulled down once on the load and released it, they were asked to tell the researcher a modified Borg scale number that corresponded to the perceived heaviness of the weight. A practice trial, including heavy and light loads, was first administered with cues of “heavy” and “light,” respectively, to familiarize subjects with the task. Then, the weights were presented to the subjects in three trials in a randomized block order. Each of the five weights was presented three times in the first two trials and twice in the third trial. Thus, each subject estimated each weight and no-weight 10 times. Subjects were given a 5–10-minute break between each trial.

### 2.6. Sound Magnitude Estimation

 Subjects were seated conformably in a sound isolated chamber and were given a set of headphones to wear. The headphones were connected to a laptop computer, which was hidden from the subjects' view. A single tone of 77, 81, 87, 92, and 96 decibels was used for sound magnitudes. The investigator was seated in the room with the subjects during the experiment. After the subjects were presented a tone, they were asked to tell the researcher a modified Borg scale number corresponding to the perceived tone loudness. A practice trial was first given with cues of “loud” and “soft” to familiarize subjects with the range of tone levels. Then, the tones were presented to the subjects in three trials in a randomized block order. Each magnitude of tone was presented three times in the first two trials and twice in the third trial. Thus, each subject estimated each sound a total of 10 times. Subjects were given a 2–5-minute break between each trial.

### 2.7. Light Magnitude Estimation

 Subjects were seated conformably in a sound isolated chamber and were asked to sit on the edge of the chair. A box fashioned into a wide, rectangular tube was used to block extraneous light from the room. One end of the box was placed around a computer monitor and the other end of the box was placed around the subjects' head and on the subjects' shoulders. A rod was placed under the box so subjects did not hold the weight of the box. A cloth was draped around the subjects to block out ambient light. The computer monitor was connected to a laptop computer, from which the experiment was run and which was hidden from subjects' view. A PowerPoint display using gray circles of 284, 192, 150, 77, and 41 lumens on a black background was used as light magnitudes. Lumens were converted to percent grey scale (83.922, 69.804, 41.176, 24.706, and 2.745, resp.) which was the magnitude scale used for analysis. The investigator was seated in the room with the subjects during experimentation. After subjects had been presented a circle of light, they were asked to tell the researcher a modified Borg scale number that corresponded to the perceived lightness of the circle. A practice trial was first given with cues of “light” and “dark” to familiarize the subject with the grayscale levels. Then, the grayscale circles were presented to the subject in two trials in randomized block order. Each magnitude of grayscale was presented five times in each trial. Thus, each subject was presented each visual grayscale a total of 10 times. Subjects were given a 2–5-minute break in between each trial.

### 2.8. Statistical Analysis

 The outcome measure for the five perception modalities was Borg scale ME as a function of modality magnitude. For all modalities, the Borg scale ME results were averaged for each modality magnitude. For resistive respiratory loading, the slope was determined by plotting Borg scale (ME) against resistive (*R*) load on a log ME/log *R* scale. The slope for pressure threshold (PT) respiratory loading was determined on a log ME/log PT load scale. Weight ME slope was determined by plotting Borg scale against weight on a log ME/log ounces scale. For auditory ME slope, the mean Borg scale for each sound intensity was plotted versus the corresponding decibel (dB) on a log ME-dB scale. Visual ME slope was found by plotting the estimated grayscale on a log ME/log gray scale plot.

 If a stimulus was given a Borg scale rating of zero on more than 5 presentations, then that stimulus was considered undetected and not included in the regression analysis. The slope was determined by linear regression analysis, and the average slope was determined for each group. The age distribution was the same for all 3 groups. All groups had age averages and ranges that are not significantly different. Overall range is 11–25 years. This raised the issue of combining the “children” (11–18 yrs) with “adults” (19–25 yrs). A Pearson correlation analysis was performed to determine if there was a significant correlation between age and modality slope. An ANOVA was used to test for group-by-modality differences followed by a Tukey's post hoc analysis. Significance was set at *P* < 0.05.

## 3. Results

 There were no significant differences in age, gender, or race between the three groups and no significant difference in severity of asthma between the asthma control group, A, (subjects without a history of life-threatening asthma) and LTA group. All subjects inspired to their target line for each test breath for magnitude estimation of resistive and pressure threshold inspiratory loads. This indicates that each subject was adequately presented each load and was able to perform the task. There was no significant difference between age and perceptual measure thus, the results for children (age 11–18) and adults (age 19–25) were pooled.

**Figure 1:**
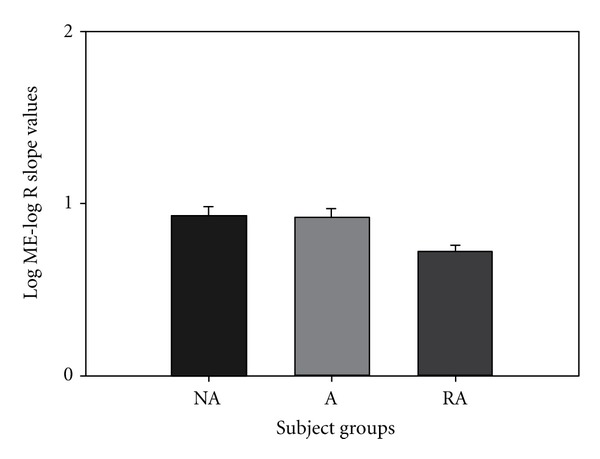
Resistive load magnitude estimation was significantly lower (*P* < 0.05) for LTA subjects than for both NA and A groups. There was no significant difference between the NA and A groups.

 The log ME/log *R* slopes for inspiratory resistive loading were 0.926, 0.921, and 0.726 for NA, A, and LTA groups, respectively. There was no significant difference between the NA and A groups for the group mean slope magnitude estimation of inspiratory resistive loads (Figure 1). The LTA subjects' resistive load magnitude estimation was significantly lower (*P* < 0.05) than the group mean slope magnitude estimation for both NA and A groups (Figure 1). There was a significant (*P* = 0.05) group effect for log ME/log *P*max for *R* load slopes. The log ME/log *P*max *R* slope for LTA subjects was significantly less than the NA and A groups.

**Figure 2:**
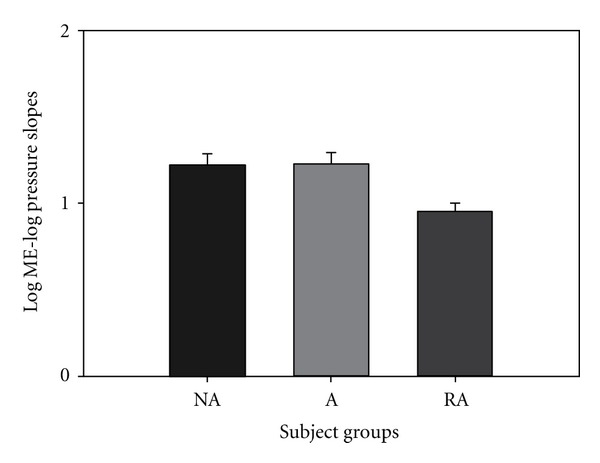
There was no significant difference between the slopes for the 3 groups, NA, A, and LTA, for inspiratory PT load ME.

 There was no significant difference between the slopes for the 3 groups, NA, A, and LTA, for inspiratory PT load ME (Figure 2). The log ME/log PT slopes for this modality were 1.224, 1.213, and 0.954 for the NA, A, and LTA groups, respectively. There was also no significant difference between the 3 groups for the PT log ME/log *P*max slopes.

**Figure 3:**
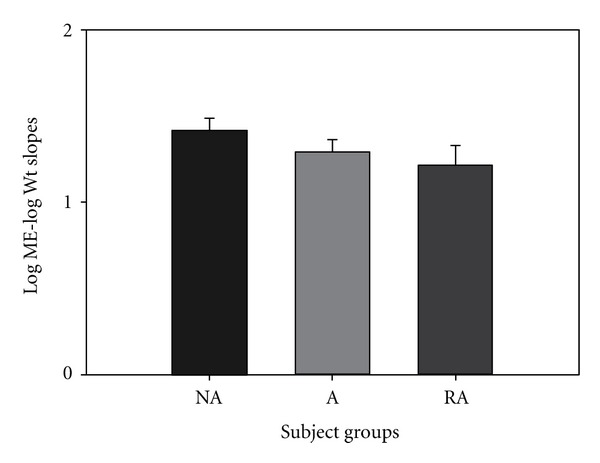
There was no significant difference between the slopes for the 3 groups, NA, A, and LTA, for weight ME on log ME/log ounces scale.

 Weight magnitude estimation slopes on a log ME/log ounces scale resulted in values of 1.414, 1.285, and 1.214 for NA, A and LTA groups, respectively, (Figure 3). None of these values reached statistical significance.

**Figure 4:**
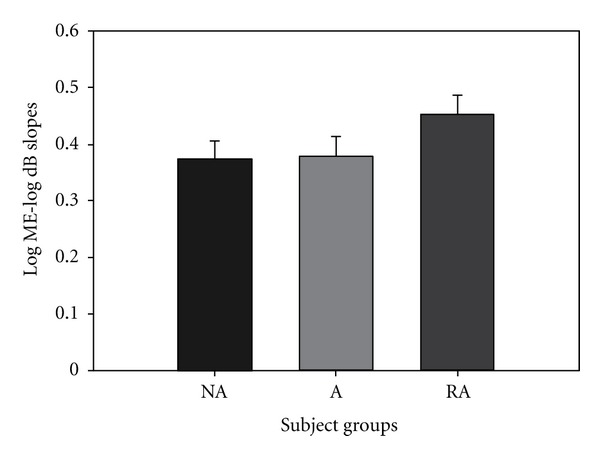
There was no significant difference between the slopes for the 3 groups, NA, A, and LTA, for auditory magnitude estimation testing.

 There was no significant difference between any of the group in auditory magnitude estimation testing (Figure 4). Slope values were 0.373, 0.379, and 0.452 for NA, A, and LTA groups, respectively.

**Figure 5:**
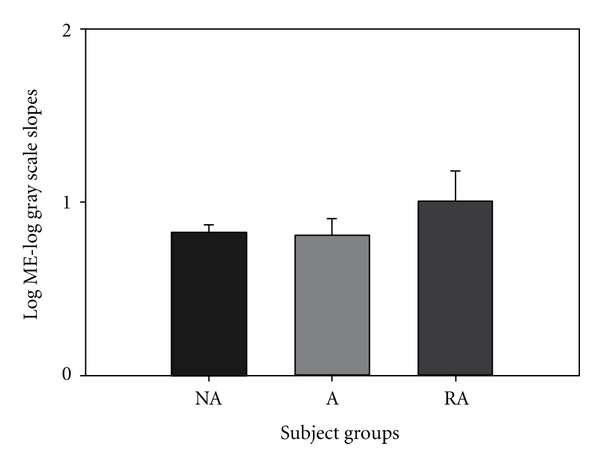
There was no significant difference between the slopes for the 3 groups, NA, A, and LTA for visual grayscale magnitude estimation testing.

 Visual gray scale magnitude estimation was plotted on a log Borg/log gray scale plot. The average slope values for this plot were 0.787, 0.810, and 1.009 for NA, A, and LTA groups, respectively, (Figure 5) and were not significantly different.

## 4. Discussion

 The perceptual sensitivity to resistive and pressure threshold respiratory loads, weight heaviness, sound intensity, and light grayscale contrast were determined for LTA, A, and NA subjects. Similar to previous reports [2, 4], LTA subjects were poor perceivers of inspiratory resistive loads, which was evidence by the significantly decreased log ME/log *R* slope in LTA subjects compared to A and NA subjects. LTA patients had a reduced perceptual sensitivity to inspiratory resistive loads but normal perceptual sensitivity to all other sensory modalities. LTA patients did not exhibit a general respiratory perception deficit, a general somatosensory deficit nor an overall sensory deficit. While the LTA subjects are poor perceivers of inspiratory resistive loads, this deficit in respiratory perception is specific to inspiratory resistive loads and is not due to perceptual deficits in other sensory modalities.

 Asthmatics with LTA are a high-risk asthmatic group due to their poor perception of respiratory resistive loads. If a patient does not feel respiratory loads during an acute asthma attack, they will be less likely to treat their condition with rescue medication. The present study demonstrated that LTA asthmatics' perception deficit is specific to only resistive respiratory loading. We did not find a significant decrease in the LTA group's magnitude estimation of pressure threshold (Figure 2), weight (Figure 3), auditory (Figure 4), or visual loads (Figure 5). Mean slope magnitude estimations for NA, A, and LTA groups for weight were comparable to previously reported values [31, 32]. Group mean auditory magnitude estimation slopes were comparable to previous studies, where slope of sound magnitude estimation versus tone level (in dB) was an average of 0.36 [26] and 0.292 [34]. We observed that our mean slope magnitude estimation of 0.373–0.452 was comparable to previously reported values. Visual gray scale measurements were slightly lower than previously reported studies [25], but this may be due to the previous study measuring reflectance rather than relative gray scale.

 If LTA asthmatics had an overall sensory deficit, we would have observed a reduced mean magnitude estimation slope of all modalities we studied. Since we did not observe a reduced mean slope, it is suggested that LTA asthmatics do not have an overall sensory deficit. Alternatively, if this unique group of patients had a somatosensory deficit, we would have observed a reduced perception of both respiratory load protocols and weight sensation. Again, we did not observe such a phenomenon, so, it is suggested that these patients do not have an overall somatosensory deficit. If the LTA group had a general respiratory perception deficit, we would have observed a reduced perception of magnitude estimation of both resistive and pressure threshold loads. Since we only observed a significant decrease in perception in resistive respiratory loads, we suggest that this group of subjects does not have an overall respiratory deficit. This study shows that LTA asthmatics have a specific deficit only in perception of resistive respiratory loads. This finding is consistent with our previous findings of reduced perception in resistive respiratory load magnitude estimation [2, 4].

 These findings are also consistent with the nature of the dangers presented to LTA asthmatics. These subjects have been hospitalized for an acute asthma attack. The perception of extrinsic resistive respiratory loads has been shown to be comparable to intrinsic respiratory loads [4]. We chose to use extrinsic respiratory loads in the present study since they are an easier and less obtrusive manner of determining resistive perception. This resistive loading mechanism is comparable to one of the many asthma symptoms that occur during an acute attack. Extrinsic resistive respiratory loading provides us a tool to observe and measure respiratory perception that correlates to some acute asthma symptoms. Pressure threshold loading produces a different sensation that is not directly related to asthma symptoms. Naturally, weight, auditory, and visual load perception have little relevance in an acute asthma attack. Thus, the only LTA subject perception deficit found in this study was that directly related to asthma symptoms.

 This study shows that LTA asthmatics have a perception deficit that is specific for some of the symptoms of an acute asthma attack. This is an important observation because LTA asthma places these patients at an increased risk for hospitalization or death due to asthma. Not perceiving their respiratory distress during an acute asthma attack and therefore decreasing likelihood of timely treatment with rescue medication often cause this increased risk. Identifying the extent of LTA asthmatics' perception deficit is an important step in developing methods and approaches to treating this unique group of patients to better manage their disease.
